# Spatiotemporal patterns of throwing muscle synergies in yips-affected baseball players

**DOI:** 10.1038/s41598-024-52332-9

**Published:** 2024-02-01

**Authors:** Toshiyuki Aoyama, Kazumichi Ae, Takahiro Taguchi, Yuna Kawamori, Daisuke Sasaki, Takashi Kawamura, Yutaka Kohno

**Affiliations:** 1 Department of Physical Therapy, Ibaraki Prefectural University of Health Sciences, 4669-2 Ami, Ami-Machi, Inashiki-gun, Ibaraki Japan; 2 Faculty of Sport Science, Nippon Sport Science University, Tokyo, Japan; 3 Graduate School of Comprehensive Human Sciences, University of Tsukuba, Tsukuba, Japan; 4 Institute of Health and Sport Sciences, University of Tsukuba, Tsukuba, Japan; 5 Centre for Medical Sciences, Ibaraki Prefectural University of Health Sciences, Ami, Japan

**Keywords:** Motor control, Neuronal physiology, Sensorimotor processing, Experimental models of disease

## Abstract

“Yips” are involuntary movements that interfere with the automatic execution of sports movements. However, how the coordination among the various muscles necessary for sports movements is impaired in athletes with yips remains to be fully understood. This study aimed to assess whether muscle synergy analysis through non-negative matrix factorization (NMF) could identify impaired spatiotemporal muscle coordination in baseball players with throwing yips. Twenty-two college baseball players, including 12 with and 10 without yips symptoms participated in the study. Electromyographic activity was recorded from 13 ipsilateral upper extremity muscles during full-effort throwing. Muscle synergies were extracted through NMF. Cluster analysis was conducted to identify any common spatiotemporal patterns of muscle synergies in players with yips. Whether individual players with yips showed deviations in spatiotemporal patterns of muscle synergies compared with control players was also investigated. Four muscle synergies were extracted for each player, but none were specific to the yips group. However, a more detailed analysis of individual players revealed that two of the three players who presented dystonic symptoms during the experiment exhibited specific patterns that differed from those in control players. By contrast, each player whose symptoms were not reproduced during the experiment presented spatiotemporal patterns of muscle synergies similar to those of the control group. The results of this study indicate no common spatiotemporal pattern of muscle synergies specific to the yips group. Furthermore, these results suggest that the spatiotemporal pattern of muscle synergies in baseball throwing motion is not impaired in situations where symptoms are not reproduced even if the players have yips symptoms. However, muscle synergy analysis can identify the characteristics of muscle coordination of players who exhibit dystonic movements. These findings can be useful in developing personalized therapeutic strategies based on individual characteristics of yips symptoms.

## Introduction

Sports movements require the coordinated behavior of numerous muscles. In athletes, “yips” is a condition of impaired muscle activity coordination^1,2^. Yips is defined as a psychoneuromuscular disorder characterized by involuntary movements that interfere with the automatic execution of fine movements^3,4^. Several studies have reported that yips occur in various sports, including golf^1,3^, cricket^4^, baseball^5,6^, archery^7^, and tennis^8^. Yips occurrence in athletes can adversely affect their careers. Yips symptoms must thus be properly assessed and treated^9,10^.

Several studies have utilized electromyography to examine the motor symptoms exhibited by athletes experiencing yips. For example, Adler et al.^11^ evaluated the activities of the forearm muscles of golfers during a putting action. They found that even in a low-pressure indoor setting, some golfers exhibited yips symptoms with co-contraction of the forearm muscles. In addition, Stinear et al.^2^ classified golfers with yips symptoms into two subtypes: focal dystonia (Type I), which primarily exhibits movement-related symptoms, and choking (Type II), which exhibits anxiety-related symptoms. Through an examination of the muscle activity of the forearm and upper arm muscles during the putting motion, they found that the forearm muscle activity was significantly higher in Type I yips even in the absence of external mental pressure. As described above, muscle activity during the putting action of golfers with yips has been clarified to some extent. However, the aforementioned studies focused on the activity of a limited number of muscles without fully elucidating whether the coordination among the multiple muscles required for sport movement would be impaired in athletes with yips.

Muscle synergy refers to a group of muscles organized to achieve a particular movement and is considered a mechanism for simplifying highly redundant human motor behavior^12^. Muscle synergy analysis was proposed as a promising method to evaluate the coordination of multiple muscles^13^. Thus, investigating muscle synergy in athletes with yips can help assess their muscle coordination disorders. Muscle synergy analysis using non-negative matrix factorization (NMF)^13–15^ decomposes the activities of a group of muscles into two, i.e., a muscle synergy vector (spatial pattern) and a synergy activation coefficient (temporal pattern). The muscle synergy vector refers to the relative contribution of each muscle within a specific synergy or motor module, whereas the synergy activation coefficient represents the temporal activation or timing of each muscle synergy. Several previous studies used muscle synergy analysis to reveal the spatiotemporal muscle activity coordination in various sports movements, such as running^16^, swimming^17^, and gymnastics^18^.

Muscle synergy analysis is also employed to describe the neuromuscular dysfunction of patients suffering from various neurological disorders. For example, several studies have demonstrated that the number of motor modules activated during walking is reduced in patients with stroke and spinal cord injury compared with healthy individuals^19,20^. The reduction in the number of motor modules was thought to be a result of the merging of motor modules that typically serve distinct functions, potentially leading to aberrant co-contraction of specific lower limb muscles^21,22^. In addition, muscle synergy analysis has revealed aberrant temporal patterns of muscle activation during walking among patients with neurological disorders such as stroke and multiple sclerosis^23–25^. These findings suggest that muscle synergy analysis can be a powerful tool for assessing spatiotemporal impairments in muscle activities induced by neural disorders. Only one study has used muscle synergy analysis to evaluate muscle coordination in athletes with yips^26^. Their study identified impairments in the temporal muscle synergy pattern in golfers with putting yips. However, muscle synergy characteristics in athletes with yips in sports other than golf, such as baseball throwing motion, have not yet been elucidated. Muscle activity and spatiotemporal muscle synergy patterns exhibit marked differences between baseball throwing motion, which requires dynamic and coordinated movements of the entire body^27,28^, and the putting motion in golf, which requires precise and relatively small limb movements^2,11,26,29^. Therefore, impaired muscle synergy characteristics in throwing yips might differ from those in putting yips. This study aimed to identify common muscle synergy spatiotemporal pattern in the throwing motion of baseball players with yips.

Golfers with yips exhibit various symptoms (e.g., freezing, jerks, jitters, and twitches)^1,3^. Focusing on the relationship between these symptoms and muscle synergies, exhibited by individual players with yips, would thus be necessary. The occurrence of yips symptoms is considerably influenced by mental pressure and playing situation^3,6^. This may be one of the factors that contribute to the difficulty of an objective diagnosis of the yips. Furthermore, yips symptoms may not always be consistently reproduced during an experimental setting that differs from the actual playing environment^11^. However, it is unclear whether muscle coordination in movements that did not reproduce the symptoms is actually preserved in athletes with yips. If muscle coordination would be impaired in athletes with yips, even in situations where their symptoms are not reproduced, muscle synergy analysis could be of significant importance in yips diagnosis. Based on these aspects, the secondary objective of this study was to determine whether the presence or absence of reproduction of yips symptoms and their characteristics during the experiment affect the spatiotemporal patterns of muscle synergy by comparing individual yips players with a control group and using cluster analysis. Given the variety of yips symptoms, we hypothesized that specific clusters or individual-specific spatiotemporal patterns of muscle synergy based on the characteristics of yips symptoms might be extracted rather than obtaining group differences between the yips and control groups.

## Methods

### Participants

**Figure 1:**
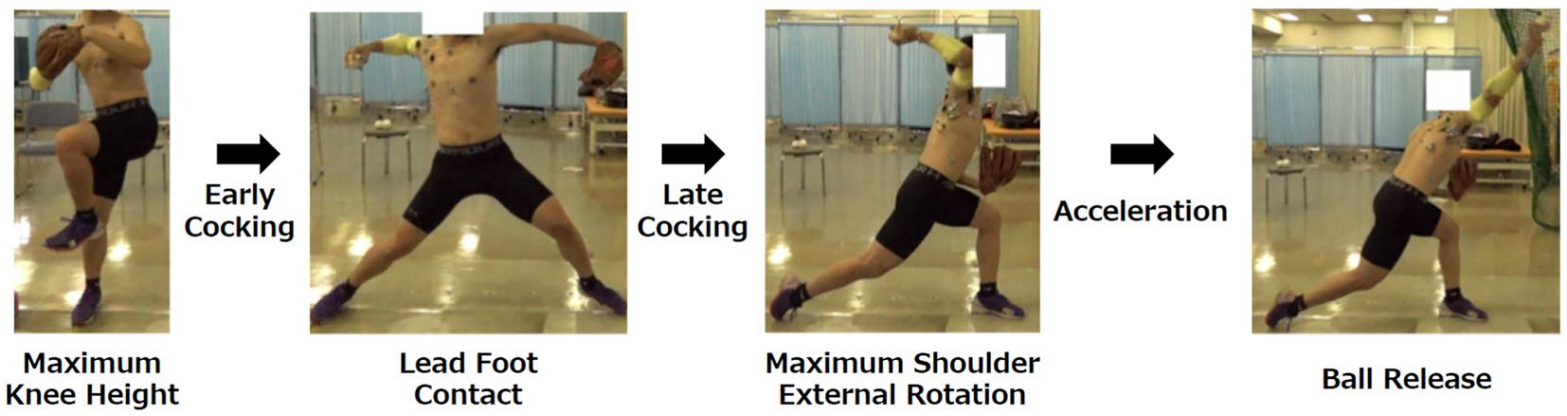
Throwing motion. Throwing motion was categorized into early cocking, late cocking, and acceleration phases.

A questionnaire survey^6,30^ regarding the presence or absence of yips symptoms was administered to players in the baseball team of the university to which the authors of this study belong. Twelve players meeting the below-described yips criteria and ten players having never experienced yips were included in this study. No player with yips received any medical treatment for their symptoms. The data of the control group in this study were obtained from a previous study^28^. Their average (standard deviation [SD]) age was 20.2 (1.2) years (yips group: 20.6 [1.4], control group: 19.8 [0.9]). The participants were exclusively male and free of any orthopedic or neurologic impairment. The average (SD) experience of the participants in playing baseball was 12.0 (1.5) years, which ranged from 10 to 14 years (yips group: 12.2 [1.6], control group: 11.9 [1.4]). The duration of their baseball experience was standard for Japanese college baseball players. The definition of yips was the same as that used in our previous studies^6^. Specifically, we defined players with yips if they met the following criteria: suddenly or gradually developing difficulties in controlling the throwing of the ball, with symptoms lasting > 1 month and continuing at the time of the experiment. Conventionally, yips is classified as Type I (Focal dystonia) or Type II (choking), characterized by physical symptoms or psychological symptoms, respectively^3,31^. Clarke et al.^32^ proposed including Type III to this classification, bearing the characteristics of both Type I and II yips. Therefore, we classified each player with yips of Type I, II, or III based on their subjective and objective symptoms. Table 1 summarizes the characteristics of players with yips. All participants gave written informed consent to participate in this study and to publish the present results as an article before their inclusion in the study. In addition, we obtained informed consent from one participant to publish the images of his throwing motion with his face cropped out (Fig. 1) in an online open access publication. All the methods of this study were carried out in accordance with the Declaration of Helsinki, and the study protocols were approved by the ethics committee of the Ibaraki Prefectural University of Health Sciences (Approval no. 926).

**Table 1:** Player characteristics in the yips group.

Player No	Age	Position	Throwing side (R/L)	Baseball experience (Year)	Duration of yips (Month)	Type of yips
Y-1	21	P	R	13	84	I
Y-2	19	C	R	11	24	II
Y-3	19	1B	R	11	26	II
Y-4	20	C	R	12	15	II
Y-5	22	LF	R	13	82	II
Y-6	23	LF	R	13	96	III
Y-7	22	SB	R	15	98	I
Y-8	19	1B	R	10	60	II
Y-9	21	C	R	14	9	I
Y-10	21	RF	R	13	18	III
Y-11	21	C	R	11	2	II
Y-12	19	LF	R	10	50	I

*P* pitcher, *C* catcher, *1B* first baseman, *LF* left fielder, *SB* second baseman, *RF* right fielder.

### Measurement of anxiety

The State-Trait Anxiety Inventory (STAI) was used to test whether anxiety was associated with abnormal muscle synergy during throwing^33^. The participant responded to the state anxiety questionnaires immediately before the evaluation of the throwing motion and the trait anxiety questionnaires after all measurements were completed.

### Throwing motion

The participants were allowed to engage in a self-directed warm-up routine before measurements. Reflective markers were attached to 24 body landmarks, namely, the head of the third metacarpal bone, radial styloid process, ulnar styloid process, medial and lateral epicondyle of the humerus, anterior and posterior shoulder joints and acromion of the throwing arm, head vertex, C7, T10, xiphoid process of the sternum, bilateral tragions, 10th ribs, heels, toes, and lateral and medial malleoli. The participants were instructed to throw a ball as forcefully as possible toward a target (bullseye) located 5 m away^28^. This distance was chosen as our questionnaire survey of players with yips had shown that the symptoms were significantly more likely to be reproduced in short- than long-distance throwing (e.g., ≤ 10 m rather than ≥ 50 m)^6^. Measurements were taken while each participant threw from the stretch position, without a full windup motion^34^. As described below, at least three successful trials were performed. After each throw, the participants reported whether their yips symptoms were reproduced or not. A three-dimensional motion capture system (VICON, UK, 250 Hz) was used to categorize the throwing motion into three phases^35^ (Fig. 1): early cocking (EC; from the maximum lead knee height until the foot–ground contact), late cocking (LC; from the lead foot contact until the maximum external rotation [MER] of the throwing shoulder), and acceleration (Acc; from the MER until ball release). Since EC was longer than the other two phases, it was subdivided into first and second halves (EC1 and EC2). Therefore, the throwing motion was classified into four phases: EC1, EC2, LC, and Acc. To clarify whether the players’ subjective movement abnormalities could be also detected objectively, the throwing motion was filmed using a standard video camera and a high-speed camera (frame rate, 240 fps; shutter speed, 1/500s). From the videos of each player, a neurologist with extensive experience in treating dystonia blindly determined the presence or absence of dystonic movements during the throwing motion.

### Electromyography

**Figure 2:**
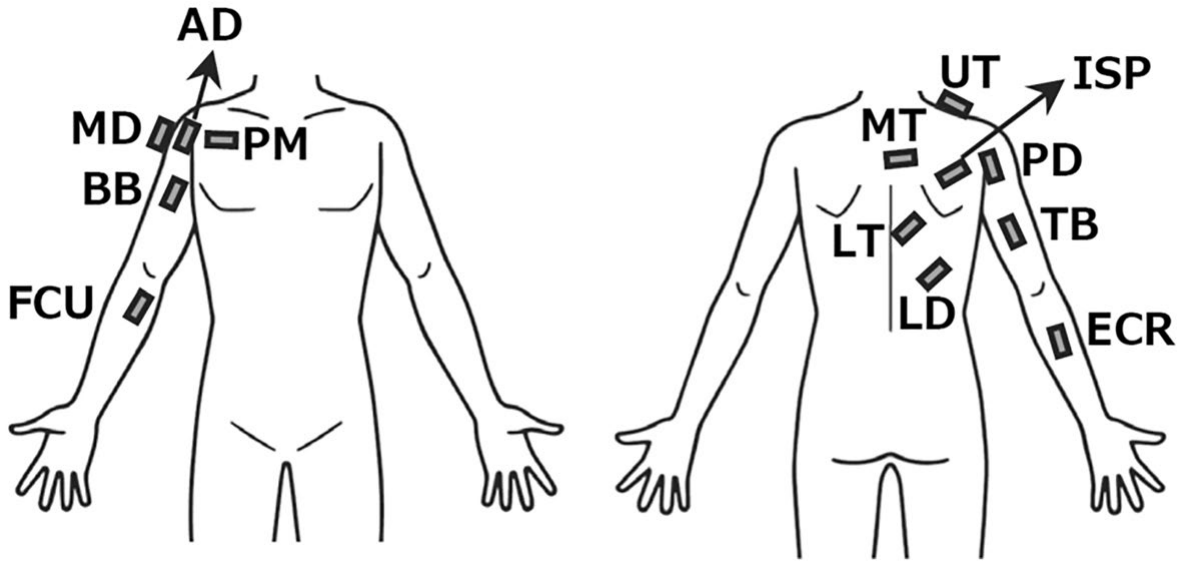
EMG electrode locations. UT: Upper trapezius, MT: Middle trapezius, LT: Lower trapezius, ISP: Infraspinatus, LD: Latissimus dorsi, PM: Pectoralis major, AD: Anterior deltoid, MD: Middle deltoid, PD: Posterior deltoid, BB: Biceps brachii, TB: Triceps brachii, FCR: Flexor carpi radialis, ECR: Extensor carpi radialis.

Electromyographic (EMG) data were collected using a wireless surface EMG system (Delsys Inc., USA) from the 13 upper limb muscles, similar to our previous study investigating muscle synergies during the baseball throwing motion^28^. The skin of the area where the EMG sensors were to be attached was shaved and rubbed with alcohol-soaked pads. After these skin preparations were ensured, EMG sensors were fixed to the upper, middle, and lower trapezius (UT, MT, and LT, respectively), infraspinatus (ISP), latissimus dorsi (LD), pectoralis major (PM); anterior, middle, and posterior deltoid (AD, MD, and PD, respectively); biceps brachii (BB); triceps brachii (TB); flexor carpi radialis (FCR), and extensor carpi radialis (ECR) muscles^36,37^ (Fig. 2). During the experiment, we verified whether the EMG activity in each trial included motion artifacts associated with rapid movement. If motion artifacts could be visually identified, additional trials were recorded. As a result, an average of 6.2 throwing trials was required to obtain three trials without motion artifact contamination. After recording, the EMG signals of these 13 muscles were high-pass filtered (zero-lag fourth-order digital Butterworth filter, cutoff frequency of 40 Hz)^38^, full-wave rectified and smoothed using a low-pass filter (zero-lag fourth-order digital Butterworth filter, cutoff frequency of 15 Hz)^39^. The filters were generated using LabVIEW software (National Instruments, TX, USA). For each trial, the duration of the throwing motion, defined as the time elapsed from the beginning of EC to ball release, was normalized to 201 data points (0–100% throwing cycle) by using a cubic spline interpolation. Finally, the EMG amplitude of each muscle was normalized to the peak amplitude per trial, and the results were averaged across the three trials.

### NMF

**Figure 3:**
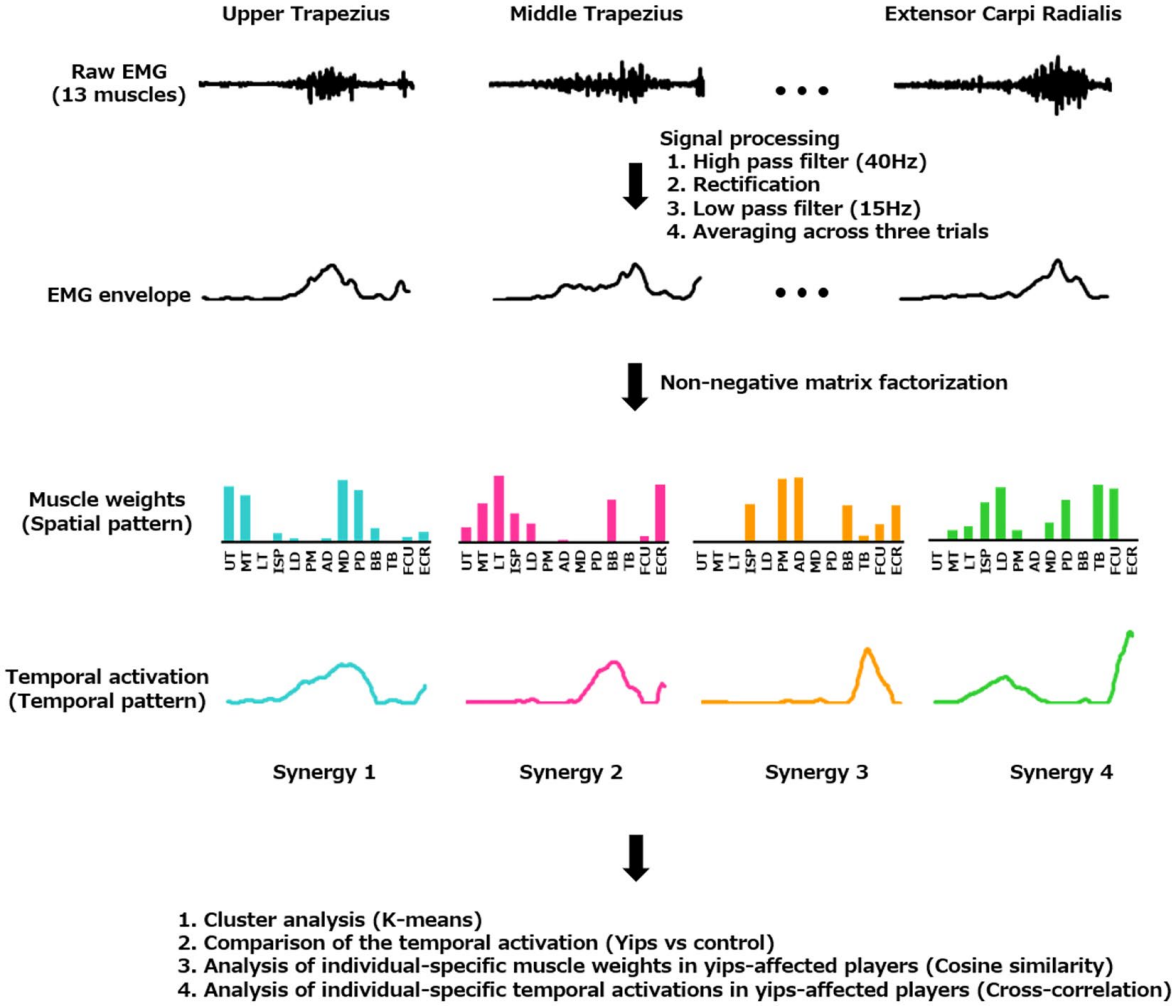
Signal processing procedure.

Complex muscle activity patterns composed of multiple muscle activities can be decomposed into low-dimensional spatial and temporal patterns by muscle synergy analysis^40^. Herein, the average EMG activity of each muscle was normalized to unit variance to equal weight variability for muscle synergy extraction. The motor modules were extracted using MATLAB (Statistics and Machine Learning Toolbox, MathWorks, Inc., USA). Specifically, NMF decomposes the non-negative EMG matrix (M) composed of 13 rows (1 per muscle) and 201 columns (1 per time point) into a linear combination of time-invariant components W_i_ with time-variant activation weights^41^ (Fig. 3) such that

$${\text{M}} = {\text{c}}_{{1}} {\text{W}}_{{1}} + {\text{c}}_{{2}} {\text{W}}_{{2}} + \cdots {\text{c}}_{{\text{n}}} {\text{W}}_{{\text{n}}} .$$

where W_i_ is a vector representing the spatial pattern of EMG activity conferred by the muscles of the *i*th motor module and thus indicates the relative contribution (between 0 and 1) of each muscle to the motor module. The time-series activation coefficient ci indicates the putative temporal neural activation pattern for the motor module Wi. The number of motor modules (n) ranging from 1 to 12 was extracted to determine the optimal number of motor modules needed to explain the original EMG waveform during the throwing motion. The NMF algorithm was iterated until either the squared error between the original and reconstructed EMG waveforms was less than 10^−6^ or the iteration number reached 500^42^. This procedure was performed 20 times to prevent local minima, and the solution with the lowest error was selected^43^. The goodness of fit between the measured and reconstructed data for each number of modules was calculated as the variance accounted for (VAF)^44^. Previously, we reported that the optimal number of motor modules during throwing motion in college baseball players was four^28^. To ascertain the suitability of the four identified motor modules for individuals experiencing yips, the Smirnov–Grubbs test was conducted on the VAF values of the four extracted motor modules, derived from all participants.

### Cluster analysis

To determine whether there were specific motor modules depending on the presence or absence of yips symptoms and their characteristics, k-means clustering was used to classify all motor modules obtained from all individual players, including the yips and control groups, based on their respective muscle weights^45^. The number of iterations was set to a maximum of 50^46^. Given the sensitivity of the k-means algorithm to the initial clustering centroid, the procedure was run 10 times with different initial centroids^46^. To determine the most appropriate number of clusters, silhouette scores were calculated for various k-values^43^, and the k-value producing the highest average silhouette score was considered optimal.

### Analysis of the temporal activation

To determine whether the timing of activation of muscle synergy, i.e., temporal activation pattern, differed between groups, the areas under the curve of the temporal activation (activation area) in EC1, EC2, LC, and Acc phases were computed as a proportion (%) of the entire activation area^25,28^.

### Analysis of individual-specific muscle synergies in yips-affected players

We examined whether the spatiotemporal pattern of muscle synergies for each player with yips deviated from the typical muscle synergy pattern of the non-yips population. Cosine similarity is the inner product of the two paired muscle-weighting vectors normalized to a unit norm. The cosine similarity is represented as the cosine of the angle between vectors, with values closer to one indicating greater similarity. The equation for the cosine similarity is as follows:

$$cosine\;similarity = \frac{{\mathop \sum \nolimits_{j = 1}^{n} A_{j} B_{j} }}{{\sqrt {\mathop \sum \nolimits_{j = 1}^{n} A_{j}^{2} } \sqrt {\mathop \sum \nolimits_{j = 1}^{n} B_{j}^{2} } }}$$

where Aj and Bj refer to the muscle weight of the jth muscle of an individual player in the control and yips groups, respectively. For each muscle synergy, the cosine similarity between each player with yips and 10 individual players in the control group was calculated. For these 10 cosine similarity values, 95% confidence intervals were calculated using bootstrapping^43,47^ with 1000 resamplings. We subsequently computed the cosine similarity for the muscle-weighting vectors between each player in the yips group and those in the control group that could have occurred by chance and defined it as chance level. Specifically, for each muscle-weighting vector comprising each muscle synergy obtained from each individual player, we created 1000 muscle-weighting vectors by randomly shuffling the muscle order. Then, for a player with yips, we calculated the shuffled cosine similarity in all combinations (1000 × 1000) with a player from the control group. The same procedures were performed with all nine other control players to calculate 1000 × 1000 × 10 randomly shuffled cosine similarity values. We estimated 95% confidence intervals for the obtained shuffled cosine similarity values and defined the lower limit of 95% confidence intervals as the chance level. If the 95% confidence interval of the unshuffled cosine similarity was below the chance level, the motor module composition of the yips player was considered specific.

We also evaluated whether the temporal activation pattern of each player in the yips group was distinct from those in the control group. Specifically, for the similarity of the temporal activation patterns among all players in the control group, we used the MATLAB xcorr function to calculate the zero-lag cross-correlation coefficient and the lag time to achieve the maximum cross-correlation coefficient^48,49^. We then calculated the similarity of the temporal activation patterns between the control and yips individuals following the same procedure.

### Statistical analysis

All statistical analyses were performed using IBM SPSS version 23.0 (IBM Corp., Armonk, NY, USA). For all group comparisons, the significance level was set at α = 0.05. The Shapiro–Wilk test was used to test the normality of dependent variables. Accordingly, The Student t-test and Mann–Whitney U test with Bonferroni correction were employed for between-group comparisons of the STAI score and VAF values, respectively. Since the normality was not confirmed, aligned rank transform (ART) two-way analysis of variance (ANOVA)^50^ with a group factor (control vs. yips) and a muscle factor (13 muscles) or a synergy factor (4 synergies) were used for the analysis of motor module composition and temporal activation patterns, respectively. To test whether the zero-lag cross-correlation coefficient and the lag time between the control and yips individuals differed from those among the control group, Kruskal–Wallis tests were performed. The Bonferroni method was used for multiple comparisons. Results are presented as mean (SD) or median (first and third quartiles).

### Ethical approval and informed consent

All participants provided written informed consent before participation in the study in accordance with the Declaration of Helsinki, and the study protocols were approved by the ethics committee of the Ibaraki Prefectural University of Health Sciences (approval no. 926).

## Results

In this study, no significant differences in age (t [20] = 1.532, *p* = 0.141) and years of baseball experience (t [20] = 0.720, *p* = 0.480) were found between the two groups. No significant differences in state anxiety (yips: 34.1 [5.9], control: 34.5 [5.3], t [20] = 0.172, *p* = 0.865) and trait anxiety (yips: 45.2 [5.4], control: 43.5 [4.9], t [20] = 0.753, *p* = 0.460) scores were noted between the two groups. Based on the symptoms experienced in each player with yips, four were classified as Type I, six as Type II, and two as Type III. During the experiment, 3 (Y-1, Y-7, and Y-12) of the 12 players in the yips group exhibited subjective motor symptoms associated with throwing yips (Table 2). In the blind video assessment of the throwing motion, the neurologist confirmed that these three players had objective dystonic movements. The respective symptoms of players with yips are shown in Table 2. The relative lengths of the EC, LC, and Acc phases in the control group were 81.9% (80.3%, 84.3%), 14.9% (12.0%, 15.9%), and 3.5% (2.7%, 4.0%), respectively, and those in the yips group were 83.6% (80.5%, 86.4%), 13.9% (10.7%, 16.1%), and 2.8% (2.3%, 3.6%), respectively. No significant differences were found between these two groups in the relative length of each of the three throwing phases (EC, Z = 0.528, *p* = 0.628; LC, Z = 0.264, *p* = 0.821; Acc, Z = 1.319, *p* = 0.203).

**Table 2:** Symptoms of each player with yips.

Player no	Symptoms during experiment	Symptoms
Y-1	+	Involuntary arm movements during the EC phase
Y-2	−	Ball slipping from the hand in high-pressure situations
Y-3	−	Uncertain throwing motion in strong-anxiety situations
Y-4	−	Inconsistent throwing accuracy at short distances
Y-5	−	Loss of hand sensation during ball release in high-pressure situations
Y-6	−	Interrupted throwing motion in nervousness situations
Y-7	+	Excessive retraction of the throwing arm during the EC phase
Y-8	−	Inconsistent throwing accuracy at short distances or with light intensity
Y-9	−	Difficulty elevating the arm during the EC phase
Y-10	−	Involuntary thumb movements in nervous situations such as games
Y-11	−	Loss of ball control in high-pressure situations due to anxiety
Y-12	+	Temporary pause of the arm motion during the LC phase

*EC* early cocking, *LC* late cocking.

### Number of motor modules

**Figure 4:**
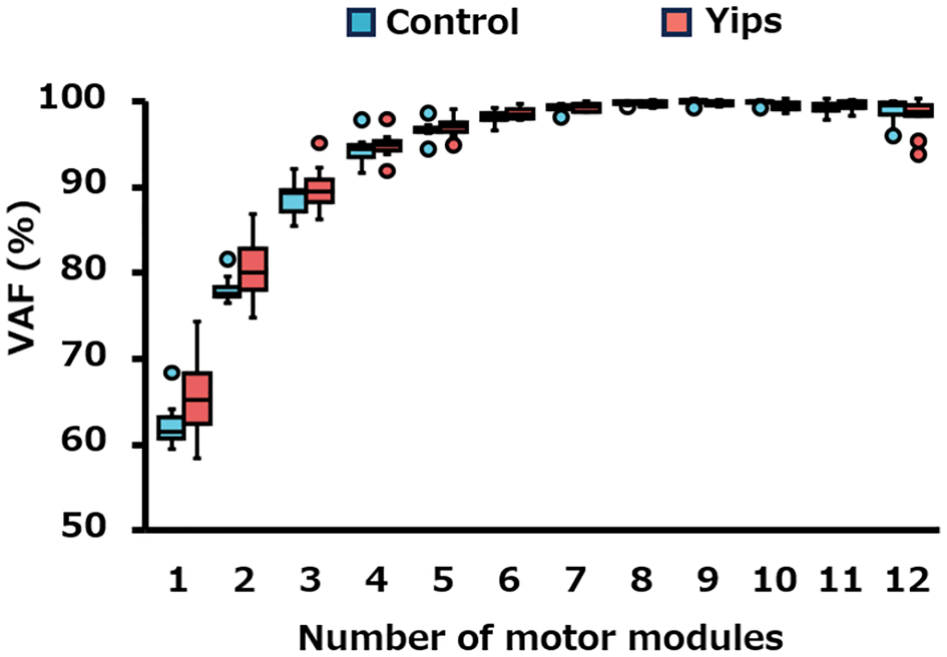
Differences in VAF values for each extracted motor modules (2–12) between the control (blue) and yips (red) groups.

Figure 4 presents the results of the VAF values for the reconstructed EMG signals using different number of motor modules. The comparisons of the VAF values between the two groups showed no significant differences in all cases where 2–12 motor modules were selected. When using four motor modules as in our previous study^28^, the VAF values for all 22 participants exceeded 90% (yips, 94.2% [93.5%, 94.9%]; control, 93.8% [92.5%, 94.2%]). In addition, the Smirnov–Grubbs test indicated the absence of outliers in the VAF values for the four motor modules. Based on these findings, the selection of four motor modules from all participants was considered appropriate.

### Composition of the motor modules

**Figure 5:**
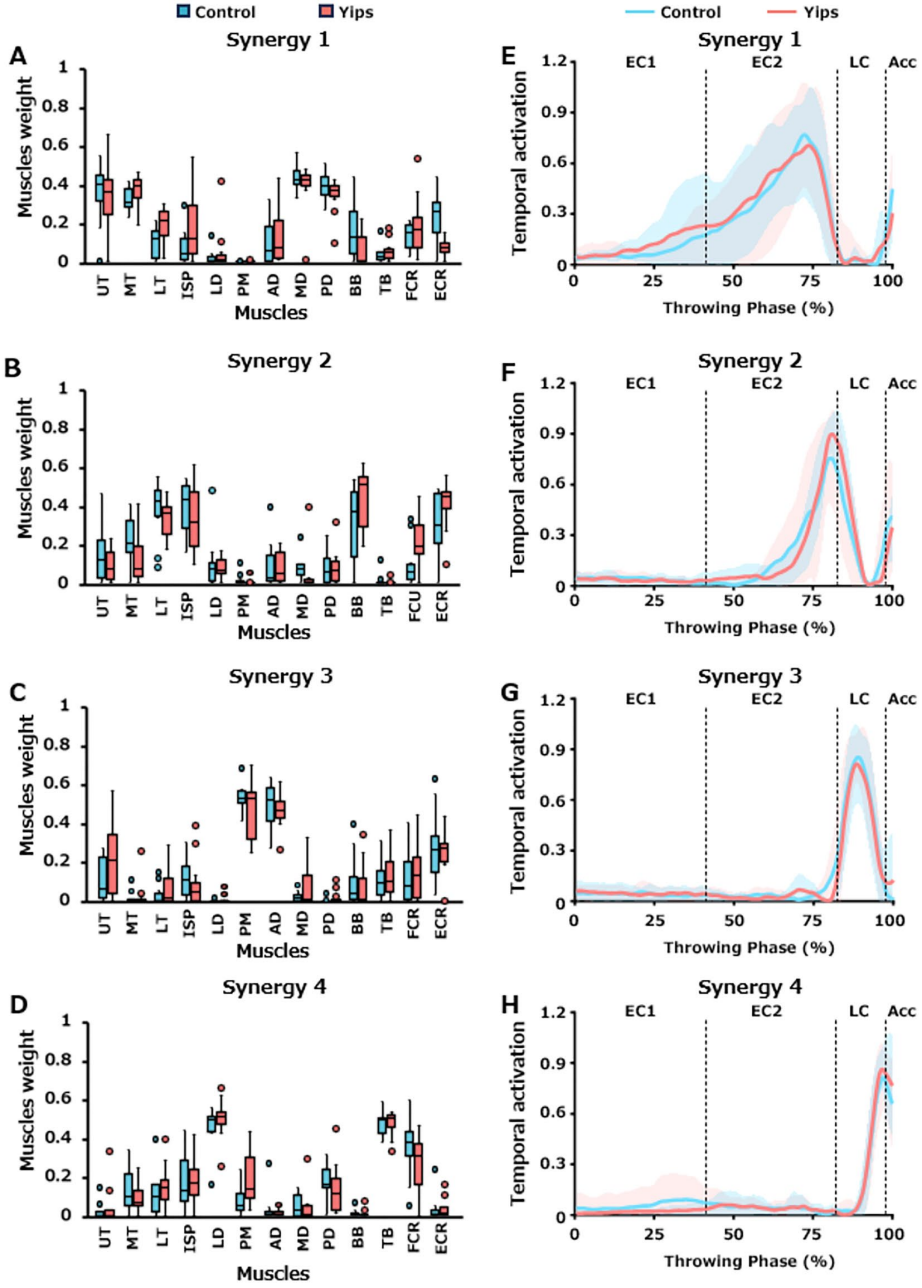
Motor module composition in each muscle synergies. Figures (**A**), (**B**), (**C**) and (**D**) show the composition (muscle weights) of muscle synergies 1, 2, 3, and 4, respectively. The blue and red box plots show data for the control and yips groups, respectively. Figures (**E**), (**F**), (**G**), and (**H**) show the temporal activation patterns of muscle synergies 1, 2, 3, and 4, respectively. The blue line and area indicate the mean and SD of the control group, respectively, whereas the red line and area indicate those of the yips group.

A total of 88 motor modules were extracted from 22 players. K-means clustering was performed to classify these 88 motor modules based on their spatial patterns. The optimal number of clusters, k, was determined to be four based on the highest silhouette scores (Supplementary Table S1). No yips-specific clusters were observed. These four clusters of muscle synergies were defined as synergies 1, 2, 3, and 4 in the order of the peak timing of each activity^28^. The composition of each of the four synergies is depicted in Fig. 5A–D. For synergy 1, a two-way ART ANOVA for the group (control vs. yips) and muscle (13 muscles) factors showed no significant interactions (F [12, 240] = 1.677, *p* = 0.072). The main effect of the group factor (F [1, 20] = 0.108, *p* = 0.746) was not significant, whereas the muscle factor (F [12, 240] = 27.867, *p* < 0.0005) was significant, with a particularly high weight of the UT, MT, MD, and PD muscles. Similarly, synergies 2, 3, and 4 showed no significant interactions (synergy 2, F [12, 240] = 1.456, *p* = 0.142; synergy 3, F [12, 240] = 0.626, *p* = 0.819; synergy 4, F [12, 240] = 1.077, *p* = 0.380) and no main effects of the group factor (synergy 2, F [1, 20] = 0.103, *p* = 0.751; synergy 3, F [1, 20] = 1.073, *p* = 0.313; synergy 4, F [1, 20] = 0.009, *p* = 0.925), whereas significant main effects of the muscle factor (synergy 2, F [12, 240] = 31.126, *p* < 0.0005; synergy 3, F [12, 240] = 29.050, *p* < 0.0005; synergy 4, F [12, 240] = 36.518, *p* < 0.0005) were obtained. In synergy 2, muscle weights were high in the LT, ISP, BB, and ECR. In synergy 3, muscle weights were higher in the PM and AD. In synergy 4, the muscle weights of the LD, TB, and FCR were particularly higher.

### Temporal activation patterns

**Figure 6:**
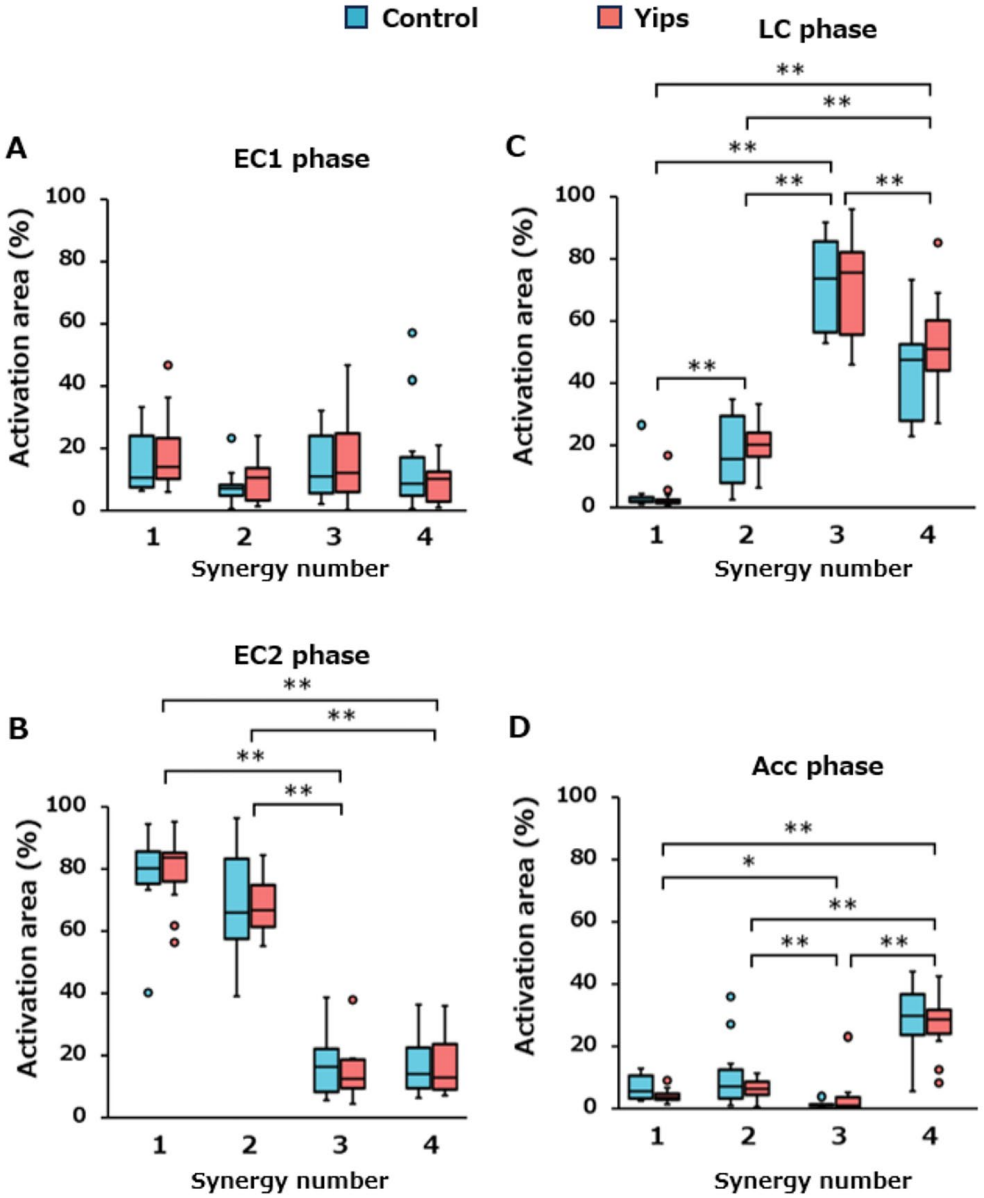
Activation areas during the EC1, EC2, LC, and Acc phases. The blue and red box plots present data for the control and yips groups, respectively.

The temporal activation of the four synergies is shown in Fig. 5E–H. In the EC1 phase, two-way ANOVA showed no significant interaction (F [3, 60] = 0.172, *p* = 0.915), and no main effect of synergy (F [3, 30] = 2.159, *p* = 0.102) and group (F [1, 20] = 0.369, *p* = 0.550) factors (Fig. 6A). In the EC2 (Fig. 6B) and LC (Fig. 6C) phases, the interaction (EC2, F [3, 60] = 0.039, *p* = 0.990; LC, F [3, 60] = 0.646, *p* = 0.589) between synergy and group factors and the main effect of the group factor (EC2, F [1, 20] = 0.092, *p* = 0.765; LC, F [1, 20] = 2.001, *p* = 0.173) were not significant, whereas a significant main effect of synergy factor was observed (EC2, F [3, 60] = 78.692, *p* < 0.0005; LC, F [3, 60] = 134.067, *p* < 0.0005). Post-hoc comparisons revealed that the activation areas of synergies 1 and 2 were significantly larger than those of synergies 3 and 4 in the EC2 phase. In addition, the activation areas were gradually larger in the descending order of synergies 1, 2, 4, and 3 in the LC phase. No significant interaction was found between synergy and group factors in the Acc phase (F [3, 60] = 0.760, *p* = 0.521). The group factor tended to be lower in the yips group, although this was not statistically significant (F [1, 20] = 4.081, *p* = 0.057). For the synergy factor, a significant main effect was obtained (F [3, 60] = 34.854, *p* < 0.0005), with synergy 3 being significantly lower than the other three synergies. Furthermore, synergy 4 was significantly higher than the other three synergies (Fig. 6D).

### Comparison between each yips player and control players

**Figure 7:**
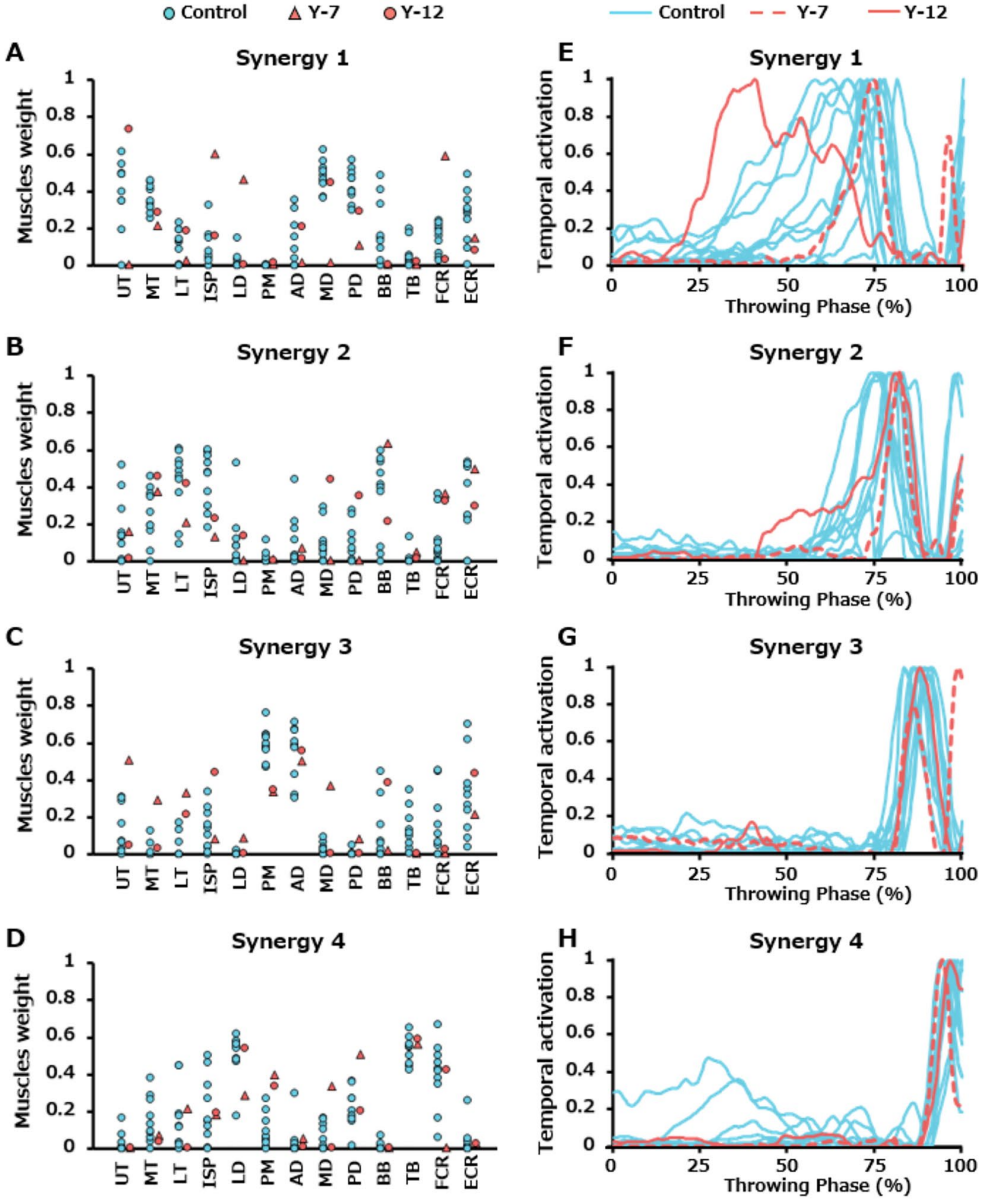
Data from two individuals for whom specific motor module compositions and temporal patterns were obtained. Figures (**A**), (**B**), (**C**), and (**D**) show the composition (muscle weights) of muscle synergies 1, 2, 3, and 4, respectively. The blue circles indicate data for each control player, whereas the red triangle and circle indicate data for players Y-7 and Y-12, respectively, who had lower spatiotemporal similarity to the control group. Figures (**E**), (**F**), (**G**), and (**H**) show the temporal activation patterns of muscle synergies 1, 2, 3, and 4, respectively. The blue lines show the data for each control player, and the red dashed and solid lines show the data for players Y-7 and Y-12, respectively.

There were two players with yips whose 95% confidence interval for cosine similarity was below the chance level. (Table 3). Figure 7 shows the motor module composition (Fig. 7A–D) and temporal activation pattern (Fig. 7E–H) of those two players and all the control individuals. Player Y-7 had a lower cosine similarity than the chance level in the motor module compositions in synergies 1, 3, and 4. Notably, synergy 1 in this player with yips had low muscle weights in the UT and MD and higher muscle weights in the ISP, LD, and FCR (Fig. 7A). In synergy 3, higher muscle weights of the trapezius and MD muscles were found, whereas a lower weight of the PM was noted (Fig. 7C). Furthermore, synergy 4 showed higher muscle weights of the PM, MD, and PD, whereas lower muscle weight of the FCR (Fig. 7D). The zero-lag cross-correlation coefficient of the temporal activation pattern (Table 4) of synergy 3 between player Y-7 and 10 control players (0.62 [0.54, 0.68]) was significantly lower than those among control players (0.74 [0.40, 0.87]) (Z = 3.799, *p* = 0.013). The temporal activation pattern of synergy 3 in the control group was characterized by unimodal activity with a peak during the LC phase. By contrast, the activity of synergy 3 in the Y-7 player exhibited a bimodal activity pattern with a peak at ball release in addition to the LC phase.

**Table 3:** Similarity of composition of muscle synergies in each player with yips.

Player no	Synergy 1			Synergy 2			Synergy 3			Synergy 4		
	Chance level	95% CI		Chance level	95% CI		Chance level	95% CI		Chance level	95% CI	
		Lower	Upper		Lower	Upper		Lower	Upper		Lower	Upper
Y-1	0.77	0.87	0.94	0.75	0.75	0.86	0.69	0.71	0.84	0.74	0.81	0.90
Y-2	0.79	0.82	0.87	0.72	0.64	0.86	0.69	0.84	0.93	0.72	0.81	0.90
Y-3	0.76	0.77	0.87	0.72	0.52	0.77	0.71	0.80	0.86	0.74	0.79	0.89
Y-4	0.78	0.72	0.81	0.73	0.60	0.75	0.71	0.73	0.85	0.72	0.79	0.88
Y-5	0.75	0.82	0.89	0.72	0.69	0.85	0.71	0.80	0.88	0.73	0.79	0.89
Y-6	0.76	0.84	0.91	0.71	0.71	0.87	0.69	0.85	0.92	0.69	0.81	0.91
Y-7	0.70	0.28	0.36	0.73	0.56	0.79	0.72	0.59	0.68	0.74	0.63	0.73
Y-8	0.76	0.70	0.80	0.75	0.77	0.88	0.72	0.66	0.74	0.72	0.71	0.81
Y-9	0.79	0.83	0.87	0.74	0.66	0.82	0.71	0.80	0.89	0.73	0.79	0.86
Y-10	0.75	0.81	0.90	0.73	0.63	0.83	0.68	0.71	0.80	0.73	0.75	0.84
Y-11	0.76	0.79	0.88	0.74	0.67	0.87	0.70	0.85	0.92	0.74	0.84	0.91
Y-12	0.74	0.73	0.87	0.76	0.62	0.74	0.70	0.68	0.84	0.72	0.81	0.91

Muscle synergies with 95% confidence interval (CI) of the cosine similarity below the chance level for muscle synergy composition are underlined.

**Table 4:** Similarity of temporal activation patterns in each player with yips.

Player no	Cluster1		Cluster2		Cluster3		Cluster4	
	Zero-lag CC	Lag time (%)	Zero-lag CC	Lag time (%)	Zero-lag CC	Lag time (%)	Zero-lag CC	Lag time (%)
Y-1	0.81 (0.50, 0.84)	1.5 (0, 8.3)	0.80 (0.69, 0.93)	3.0 (0.9, 6.1)	0.87 (0.80, 0.94)	2.0 (0.5, 3.4)	0.91 (0.81, 0.92)	0 (0, 1.4)
Y-2	0.80 (0.60, 0.91)	5.5 (3.6, 8.0)	0.80 (0.67, 0.90)	4.0 (1.1, 7.1)	0.90 (0.79, 0.96)	2.0 (0.5, 2.6)	0.85 (0.79, 0.89)	0 (0, 1.0)
Y-3	0.84 (0.48, 0.94)	3.0 (0.4, 9.6)	0.56 (0.42, 0.92)	5.3 (1.0, 8.5)	0.66 (0.52, 0.79)	3.5 (1.9, 5.6)	0.90 (0.79, 0.94)	0.5 (0, 1.5)
Y-4	0.86 (0.63, 0.93)	2.5 (0, 9.1)	0.77 (0.63, 0.88)	3.0 (0.9, 5.9)	0.84 (0.76, 0.91)	2.0 (0.5, 3.0)	0.95 (0.82, 0.96)	0.5 (0, 1.1)
Y-5	0.50 (0.24, 0.87)	12.5 (7.4, 18.0)	0.77 (0.64, 0.89)	4.8 (0.9, 7.8)	0.92 (0.82, 0.94)	1.5 (1.0, 3.3)	0.92 (0.88, 0.96)	0 (0, 0.5)
Y-6	0.85 (0.77, 0.90)	4.3 (0.8, 6.3)	0.81 (0.69, 0.92)	3.5 (1.0, 5.8)	0.89 (0.78, 0.96)	1.8 (0.9, 2.6)	0.93 (0.88, 0.95)	0 (0, 0.9)
Y-7	0.78 (0.52, 0.85)	3.0 (1.5, 8.0)	0.65 (0.49, 0.87)	4.0 (0.8, 6.9)	0.62* (0.54, 0.68)	2.5 (1.5, 3.6)	0.77 (0.68, 0.84)	2.5 (1.1, 3.1)
Y-8	0.73 (0.49, 0.96)	7.3 (0.9, 10.6)	0.85 (0.72, 0.91)	3.5 (2.0, 5.3)	0.80 (0.68, 0.84)	2.3 (1.4, 3.5)	0.72 (0.65, 0.79)	3.0 (2.1, 3.5)
Y-9	0.88 (0.54, 0.91)	2.3 (0.9, 9.0)	0.82 (0.71, 0.91)	3.0 (0.8, 6.3)	0.85 (0.68, 0.96)	2.3 (0.9, 4.0)	0.93 (0.86, 0.95)	0 (0, 0.9)
Y-10	0.86 (0.72, 0.95)	2.5 (0, 7.6)	0.64 (0.51, 0.94)	5.0 (0.4, 8.4)	0.91 (0.77, 0.96)	1.5 (0.4, 3.0)	0.95 (0.83, 0.96)	0.3 (0, 1.5)
Y-11	0.85 (0.65, 0.93)	4.8 (2.5, 6.1)	0.80 (0.61, 0.91)	3.0 (1.9, 4.6)	0.89 (0.83, 0.93)	2.0 (0.5, 3.0)	0.92 (0.87, 0.94)	0 (0, 0.5)
Y-12	0.44 (0.25, 0.77)	33.3** (10.9, 36.3)	0.81 (0.69, 0.88)	3.5 (0.9, 6.0)	0.92 (0.81, 0.95)	1.5 (0.9, 3.0)	0.92 (0.88, 0.97)	0 (0, 0.5)

Zero-lag cross-correlation (CC) and lag time to achieve the maximum CC are shown. The values are shown as medians (first and third quartiles). *p < 0.05, **p < 0.01.

Player Y-12 showed a lower cosine similarity value for the motor module composition in synergy 2 than the chance level (Table 3). Specifically, synergy 2 in this player had high muscle weights of the MD and PD (Fig. 7B). The lag time to achieve the maximum cross-correlation of synergy 1 between this player and each player in the control group (33.3% [10.9%, 36.3%]) was significantly greater than those obtained among players in the control group (6.5% [2.3%, 10.0%]) (Z = 5.301, *p* < 0.0005, Table 4, Fig. 7E).

## Discussion

The findings of this investigation revealed that four muscle synergies were extracted from the yips group that were common to the control group. Furthermore, there were no significant differences in the spatial and temporal patterns of those four synergies between the two groups. These results suggest that there are no specific muscle synergies in throwing motions that are common to players with yips. However, analysis of individual players with yips showed a specific spatiotemporal pattern of muscle synergies that deviated from the control group in two players who reproduced the dystonic symptoms during the experiment. These results suggest that aberrant muscle coordination during throwing motion in players with yips is related to the existence of dystonic symptoms.

### Differences in muscle synergies between the two groups

In studies of golfers with symptoms of “putting yips,” co-contraction of the forearm muscles was observed^11,29^. Co-contraction of multiple muscles has been proposed as a potential cause of merging of multiple muscle synergies into a single one^21,22^. However, the present study of baseball players with throwing yips did not yield any findings indicating the presence of such a merging of muscle synergies. In addition, the cluster analysis utilizing k-means revealed that no motor module was specific among players of the yips group. Furthermore, no group differences in the composition of the four obtained muscle synergies were noted**.** These results suggest the absence of a common spatial pattern of muscle synergy specific to players with yips.

As regards the temporal activity pattern of muscle synergy, although no significant differences were noted in the activation area of the four throwing phases between the two groups (Fig. 6), the yips group tended to have a lower activation area of the Acc phase as a percentage of the total activation area (*p* = 0.057). In previous studies, the Acc phase was one of the periods when various muscles were most activated in preparation for ball release^27,28^. However, previous studies demonstrated that players with yips and those with focal dystonia are characterized by excessive muscle contractions^2,51^, being inconsistent with the results of this study. Therefore, we speculate that the lower relative muscle activity levels at the Acc phase in the yips group observed in this study might reflect compensatory strategies to cope with the ball control difficulties caused by yips, rather than the yips symptoms per se. Although no conclusions could be drawn from the present results related to these aspects, determining whether the reduced muscle activity during the Acc phase in players with yips are the primary cause of poor motor performance or secondary changes would be significantly important to address their symptoms; further investigation is thus required.

To our knowledge, only one study has examined muscle synergy in players with yips. Revankar et al.^26^ compared the muscle synergies in the upper limb during the putting movement between normal and yips shots. Their study revealed no significant difference in the motor module composition between the normal and yips shots; however, a notable difference in the temporal activation patterns was found in 11 of 15 participants. Another study examined muscle synergies in keystroke movements of pianists with focal dystonia, a subtype of the yips^52^. They reported that there is no muscle coordination pattern specific to pianists with focal dystonia, and that existing muscle coordination patterns are partially altered. Accordingly, the findings in the present study, i.e., no common muscle synergies specific to yips players were identified, yet deviations from the typical spatiotemporal activity patterns of muscle synergies were observed in certain individuals, as discussed below, are consistent with the results of these previous studies.

In this study, no significant differences could be observed in trait and state anxiety between the two groups. Conventionally, anxiety is considered to be closely related to the yips symptoms. However, in support of our results, several previous studies reported that anxiety in the yips and control groups did not differ^10,53–55^. Hence, anxiety might be a factor that exacerbates yips symptoms rather than a cause of yips. In any case, this study does not allow for determining how anxiety affects muscle synergy. Elucidating how anxiety affects muscle synergy might be possible by establishing situations with and without high pressure, as in the study conducted by Stinear et al.^2^ in golfers with yips.

### Characteristics of muscle synergies in players with yips

As previously stated, between-group comparisons revealed no distinctive differences in muscle synergies during throwing. However, a more in-depth examination of the spatiotemporal patterns of muscle synergy in individual players with yips in comparison to those of the control group yielded some intriguing findings. In player Y-7, the similarity of the muscle weight classified into synergy 1 was remarkably lower than the chance level (Table 3). The motor modules of synergy 1, mainly composed of the MD and UT, typically contribute to the upper limb elevation during the EC phase. However, the weights of the UT and MD were reduced, whereas the weights of the ISP and LD were increased. This suggests that posterior upper limb elevation (shoulder extension movement) may be achieved by the LD instead of lateral elevation (shoulder abduction movement) by the UT and MD during the EC phase^28^. Thus, the distinctive composition of motor modules in this player may corroborate the subjective and objective symptoms of involuntary excessive arm retraction movements during the EC phase (Table 2). Furthermore, LD and ISP act on the internal and external rotations of the shoulder joint, respectively. Since the co-contraction of muscles with opposing actions is considered one of the characteristics of dystonic movements^11,29^, it may be related to the yips symptoms of this player. The motor module composition of synergies 3 and 4 were below the chance level (Table 3), and significant differences were noted in the zero-lag cross-correlation coefficient of synergy 3 (Table 4). We could not find any subjective or objective symptoms related to the characteristics of the spatiotemporal pattern of these muscle synergies. Thus, these symptoms are not merely yips symptoms but reflect subsequent changes in muscle coordination to compensate for abnormalities in synergy 1.

The zero-lag cross-correlation coefficients of temporal activation patterns for synergy 1 between player Y-12 and controls were significantly different from those among control players (Table 4). More specifically, the interval between the activity of synergies 1 and 2 was markedly prolonged as a result of the earlier timing of the peak activity of synergy 1 (Fig. 7E, F). In addition, the similarity of the muscle weight of synergy 2 in this player was lower than the chance level (Table 3). Previous research has revealed that synergy 2 was mainly composed of LT, ISP, BB, and ECR muscles and considered to stabilize the upper limb position, which is required in the transition from the EC to the LC phase^28^. However, in this player, the muscle weights of the MD and MT were elevated. He complained that his arm movement temporarily stopped during these throwing phases (Table 2). We consider the delayed transition of muscle activities from synergies 1–2 and the accompanying changes in the spatial pattern of muscle synergy 2 to be closely related to his symptoms.

The commonality observed in the two players with low spatiotemporal similarity of muscle synergy patterns was that subjective motor abnormalities and objective dystonic movement were identified during the experiment. Adler et al.^11^ reported that the co-contraction of forearm muscles was more likely to be observed in yips-affected golfers with dystonic movement. Their results indicating that the presence or absence of dystonic symptoms is closely related to impaired muscle coordination are consistent with the present results. Nevertheless, the literature on yips primarily focuses on measuring the level of muscle activity and co-contractions in a limited set of muscles^2,11,29^. These findings fail to provide a comprehensive understanding of the spatiotemporal muscle coordination that is critical for performing dynamic sports movements. Therefore, muscle synergy analysis may be used to investigate the characteristics of impaired spatiotemporal muscle coordination in baseball players who exhibit dystonic movements during throwing. This may have important implications for the development of personalized therapeutic strategies based on the individual characteristics of yips symptoms.

By contrast, in many players with yips, their symptoms were not reproduced during the experiment. Whether yips symptoms are reproduced strongly depends on the situation, such as mental pressure and the playing environment^3,6^. This could explain why many of the players with yips in this study did not reproduce yips symptoms during the experiment. In the present study, the spatiotemporal patterns of muscle synergies of players in the yips group, whose symptoms were not reproduced during the experiment, were similar to those of control players. This finding suggests that the spatiotemporal pattern of muscle synergies in baseball throwing motion is not impaired in situations where symptoms are not reproduced, although they have yips symptoms. Therefore, to identify impaired muscle coordination associated with throwing yips, examining each player in situations in which their symptoms are reproduced is necessary.

Given the situation-dependent variation in the presence or absence of yips symptoms, whether the intra-individual symptom changes depend on differences in the composition or the temporal activation of muscle synergies is an interesting argument. Unfortunately, because the study data were obtained in a fixed experimental setting, no athletes experienced both trials in which yips symptoms were reproduced or not during the experiment. Therefore, we cannot address whether temporal or spatial patterns of muscle synergy, or both, are involved in intra-individual variability regarding yips symptoms. Future research on this point in sports, including baseball, is necessary.

There are still no clear diagnostic criteria based on objective evaluations for yips. Therefore, as in previous studies on yips^1,3,6,7^, we identified players with yips using subjective symptoms. The fact that symptoms did not occur during the experiment in many players in the yips group may suggest not only that the symptoms vary with the situation, as previously described, but that the diagnosis of yips based on subjective assessment may overestimate its presence. Therefore, the assessment of muscle coordination using muscle synergy analysis in athletes with yips may contribute to a more objective diagnosis.

This study has some limitations. First, only 3 of the 12 players with yips reproduced their symptoms during the experiment. This may be the reason why no significant difference in throwing muscle synergies were found between the control and yips groups. It is difficult to reproduce yips symptoms that occur in baseball players in experiments conducted uniformly because the occurrence of yips depends on various factors, such as throwing distance, throwing intensity, and intensity of psychological pressure^6^. Furthermore, given that players with yips exhibited various symptoms^1,3^, it is necessary to examine muscle coordination changes that occur in situations in which symptoms are reproduced or not reproduced for each player with yips rather than comparing two groups in a fixed experimental setting. Second, in this study, we were only able to record three successful trials (an average of 6.2 trial attempts) to reduce the effect of muscle fatigue on muscle activity and the risk of developing shoulder and elbow pain associated with full-effort throwing. Therefore, we cannot exclude the possibility that recording more trials would have changed the presence or absence of yips symptom reproduction during the experiment. Third, of the three players whose yips symptoms were reproduced during the experiment, one (player Y-1) showed no distinctive spatiotemporal patterns of muscle synergies. This implies that muscle synergy analysis may not be able to explain all dystonic throwing movements reproduced during the experiment. Future studies are needed to compare yips symptoms at each muscle, as they may be caused not only by impaired spatiotemporal coordination of muscles but also by hyperactivity of certain muscles^2^.

## Conclusion

This study provides a novel finding that deviations from the typical muscle synergy pattern were observed only in players in whom dystonic movements were reproduced during the experiment. Furthermore, these spatiotemporal abnormalities in muscle coordination may explain their aberrant movements. These findings could be useful in the development of personalized treatments based on the individual characteristics of yips symptoms.

### Supplementary Information

Supplementary Information.

## Data Availability

The dataset of this study are available from the corresponding author on reasonable request.
