# Supplementary material for: Spatiotemporal patterns of throwing muscle synergies in yips-affected baseball players
Source: Sci Rep. 2024 Feb 1;14:2649. doi: 10.1038/s41598-024-52332-9 (PMC10834996; doi:10.1038/s41598-024-52332-9)
Supplement: Supplementary file 1 — Supplementary Information. [file 41598_2024_52332_MOESM1_ESM.pdf]

Supplementary Table S1. Silhouette scores for each number of clusters.

Number of clusters	2	3	4	5	6	7	8
Silhouette score	0.38 (0.14)	0.54 (0.18)	0.59 (0.24)	0.56 (0.21)	0.49 (0.25)	0.46 (0.26)	0.29 (0.24)

Mean (SD) silhouette scores are shown
